# Comparative analysis of external beam radiotherapy versus portal vein stent implantation combined with local and systemic therapy in hepatocellular carcinoma patients with portal vein tumor thrombus: a real-world retrospective study

**DOI:** 10.3389/fonc.2025.1671027

**Published:** 2025-10-06

**Authors:** Wenping Luo, Guodong Wang, Shaojun Chen, Zhe Wang, Chuang Li, Chunwang Yuan, Jingsong Mao, Wenqi Liu

**Affiliations:** ^1^ Department of Radiation Oncology, The Second Affiliated Hospital of Guangxi Medical University, Nanning, Guangxi, China; ^2^ Department of Oncology, Fourth Affiliated Hospital of Guangxi Medical University, Liuzhou, Guangxi, China; ^3^ Tumor Center of Zhongshan Hospital Affiliated to Dalian University, Dalian, China; ^4^ Liver Disease and Cancer Interventional Therapy Center, Beijing Youan Hospital, Capital Medical University, Beijing, China; ^5^ The Sixth School of Clinical Medicine, the Affiliated Qingyuan Hospital (Qingyuan People’s Hospital), Guangzhou, Guangdong, China

**Keywords:** external beam radiotherapy, portal vein 125I seed stent implantation, hepatocellular carcinoma, portal vein tumor thrombus, local and systemic therapy

## Abstract

**Objective:**

To compare the effectiveness and safety of External Beam Radiotherapy (EBRT) versus Portal Vein Stent Implantation (PVSI) when combined with local interventional therapy and TKI plus ICIs in patients with hepatocellular carcinoma (HCC) and portal vein tumor thrombus (PVTT), providing real-world evidence for clinical decision-making.

**Methods:**

This retrospective cohort study included patients with HCC and PVTT who received either EBRT or PVSI in combination with transarterial interventional therapy, TKIs, and ICIs between January 2019 and January 2025. The primary effectiveness outcomes were overall survival (OS) and progression-free survival (PFS), which were analyzed using the Kaplan-Meier method and compared using the log-rank test. Secondary outcomes included objective response rate (ORR) and disease control rate (DCR) based on mRECIST criteria. Safety outcomes were assessed by documenting the incidence and severity of procedure-related complications and drug-induced liver injury according to CTCAE guidelines. Multivariate Cox regression and pre-specified subgroup analyses were performed to identify prognostic factors.

**Results:**

This study enrolled 67 patients (26 in the EBRT group and 41 in the PVSI group) with balanced baseline characteristics and a median follow-up of 21.0 months. The EBRT group showed superior efficacy, with significantly higher 6-month objective response (38.5% vs 14.6%, P = 0.028) and disease control rates (84.6% vs 58.5%, P = 0.025). Survival analysis demonstrated a significantly longer median overall survival in the EBRT group (35 months vs 19 months, P = 0.044), while the median progression-free survival was not reached, surpassing that of the PVSI group (11 months). Multivariate analysis identified EBRT treatment (HR=2.247, 95% CI: 1.090–5.404, P = 0.030) and AFP < 400 ng/mL (HR=0.329, 95% CI: 0.137–0.791, P = 0.013) as independent predictors of overall survival. Subgroup analysis further indicated that the survival benefit associated with EBRT was particularly pronounced among patients with VP2-type portal vein tumor thrombus and those receiving TKI combined with ICIs (median OS: 36 months vs 14 months, P = 0.017; 36 months vs 12 months, P = 0.005). The adverse event profiles varied between groups: grade 1-2 leukopenia was more common in the EBRT group (46.2% vs 7.3%, P<0.001), whereas grade 1-2 aspartate aminotransferase elevation was more common in the PVSI group (70.7% vs 38.5%, P = 0.009). Although grade 3-4 toxicities were generally infrequent, hyperbilirubinemia and hypoalbuminemia occurred relatively more often (approximately 20%) in the PVSI group.

**Conclusion:**

The combination of EBRT with local interventional procedures plus TKI and ICIs significantly improved survival in HCC patients with PVTT. The median overall survival (OS) was nearly doubled compared to those not receiving this combined approach, with particularly marked benefits observed in patients with VP2-type PVTT and those receiving TKI combined with ICIs. PVTT classification, liver function, and bone marrow reserve have a significant influence on prognosis. Additionally, AFP < 400 ng/ml (P < 0.05) and EBRT (P < 0.05) were identified as critical predictors of survival. However, this combined regimen was associated with increased treatment-related toxicities, necessitating careful hematologic monitoring during treatment.

## 1 Introduction

GLOBOCAN 2023 reports 960,000 new cases of Hepatocellular Carcinoma (HCC) worldwide (623,000 in men and 337,000 in women), with an age-standardized mortality rate (ASDR) of 8.3 per 100,000. 47.1% of cases originate from China (1). Since 44% to 62.2% of patients present with Portal vein tumor thrombus(PVTT), HCC is often diagnosed at advanced stages, increasing the risk of variceal hemorrhage and preventing curative surgery (2). The median survival without treatment ranges from 2.7 to 4.0 months (3).

The Barcelona Clinic Liver Cancer (BCLC) staging system classifies hepatocellular carcinoma (HCC) with portal vein tumor thrombosis (PVTT) as BCLC-C, for which the 2022 guidelines recommend systemic therapy (4). In contrast, China’s Primary Liver Cancer Diagnosis and Treatment Guidelines (2024) advocate for combining systemic and local therapies for China Liver Cancer Staging (CNLC) IIIa/IIIb HCC with PVTT (5). Transcatheter arterial chemoembolization (TACE) and External beam radiotherapy (EBRT) are types of local therapies. Among radiotherapy options, external beam radiotherapy (EBRT) precisely targets tumors while sparing healthy tissue, making it suitable for patients with compromised liver function. Portal vein stent implantation with radioactive seeds (PVSI) enhances portal blood flow and tumor control by integrating mechanical stenting with continuous radiation (6–9). The increasing use of targeted therapies and immunotherapies has prompted numerous investigations into their combination with local treatments like TACE and radiotherapy to enhance patient survival outcomes. Current evidence indicates that combining EBRT with TKIs or TKI-ICI regimens achieves superior outcomes compared to TKI monotherapy or TKI-ICI combinations without radiotherapy (10–12). For HCC patients with PVTT, a multimodal strategy incorporating iodine-125 seed strands, portal vein stents, TACE, lenvatinib, and anti-PD-1 antibodies has demonstrated both safety and efficacy (13, 14). The comparative effectiveness of EBRT versus PVSI remains unexplored in previous research. This retrospective analysis assesses their respective safety profiles, treatment outcomes (including objective response rate, duration of response, overall survival, and progression-free survival), and the prognostic value of PVTT grading.

## 2 Materials and methods

### 2.1 This retrospective study enrolled patients with advanced hepatocellular carcinoma and portal vein tumor thrombosis treated at Liuzhou Workers’ Hospital between January 2019 and January 2025

HCC diagnosis required clinical or histological confirmation (15, 16), with staging based on the Barcelona Clinic Liver Cancer (BCLC) system (BCLC-C (17)) or China Liver Cancer (CNLC) classification (CNLC IIIa/IIIb). PVTT was assessed using Cheng’s classification in unresectable cases among patients aged 18–75 years. Eligible patients had Child-Pugh A or B liver function (18), an ECOG performance status of 0–2 (19), and PVTT confirmed by triphasic dynamic CT (20) within seven days before treatment; those unsuitable for liver transplantation or percutaneous radiofrequency ablation were included. Exclusion criteria comprised recurrent HCC, distant metastases, prior anticancer therapies (surgery or systemic treatment), Child-Pugh C status, and hepatitis C or HIV coinfection. The study received ethical approval from Liuzhou Workers’ Hospital, with waived informed consent due to its retrospective design. Patients lost to follow-up or with incomplete data were excluded. Given the retrospective nature of this study and its minimal risk to participants, the Ethics Committee of Liuzhou Workers' Hospital waived the requirement for informed consent.

### 2.2 Treatment Measures

The EBRT group received 3D-CRT or IMRT (5) at a recommended dose of 50–60 Gy. The gross tumor volume (GTV) encompassed intrahepatic PVTT and adjacent lesions, unless excluded due to minimal liver volume or high tumor burden. The planning target volume (PTV) was defined as GTV plus a 3–5 mm margin. Dose constraints included a mean liver dose of <28–30 Gy for Child-Pugh A patients and <6 Gy for Child-Pugh B patients; radiation therapy was contraindicated for Child-Pugh C cases (21, 22). Additional constraints were V5 <5% for the small bowel, V45 <45% for the stomach (maximum dose <54 Gy), spinal cord maximum <45 Gy, and mean kidney dose <15 Gy The maximum dose for both the stomach and the small intestine should be less than 54 Gy, with V for the stomach <45% and V for the small intestine ≤5%. The average dose of both kidneys is ≤15Gy. If the average dose of one kidney is greater than 19Gy, the other kidney should be avoided as much as possible. The maximum dose to the spinal cord is <45 Gy (23).

Group PVSI: (1) Preoperative planning using the TPS system involved precise delineation of the portal vein tumor thrombus target area on portal venous phase contrast-enhanced CT scans. The treatment plan used 125I seeds with 0.6-0.8 mCi activity per seed, delivering a prescribed internal radiotherapy dose of 70–150 Gy. The planned target volume achieved over 90% coverage of the tumor thrombus (V90 > 90%), with dosimetric parameters meeting D90 > 90%, BED10 > 140 Gy, and EQD2 > 80 Gy. To prevent severe complications such as radiation-induced ulcers, perforations, or fistulas, the gastrointestinal tract (including the duodenum and stomach) should receive a D1cc below 30–40 Gy, with an absolute maximum dose (Dmax) not exceeding 50 Gy. The liver V30 must remain under 30% to minimize the risk of radiation-induced liver disease. The Dmax to the portal vein wall hotspot should be constrained to 150–200 Gy to avoid vascular rupture and bleeding, while the common bile duct Dmax should be kept below 100 Gy to prevent radiation-induced stenosis. The spinal cord Dmax must not exceed 20–25 Gy, and the kidney V15 should be limited to less than 30%. (2) Seed chain preparation followed the TPS plan by loading seeds into the implant gun and connecting them to a 4F drainage catheter. The push rod sequentially advanced seeds to form a densely packed chain, confirmed under DSA fluoroscopy to span the entire tumor thrombus length. (3) The hybrid CT-DSA procedure for seed stent implantation began with CT-guided selection of an optimal percutaneous puncture route to the portal vein branch subsegment. After standard sterile preparation, an 18G coaxial needle punctured the portal vein branch, followed by guidewire exchange and 8F sheath placement. DSA guidance facilitated 6F guiding catheter insertion using a double-stiff-wire technique, with angiography confirming tumor thrombus location and guidewire positioning. A portal vein stent (88×12 mm or 100×20 mm) was deployed over the stiff wire, followed by custom seed chain delivery through the 6F catheter. Post-deployment DSA verified proper seed chain positioning within the stent lumen. An 8F balloon expanded the stent to compress the seed chain, with final angiography confirming optimal stent placement and portal vein patency. The procedure concluded with sheath removal, pressure dressing application, and 4-hour monitoring. Postoperative day 1, abdominal ultrasound and complete blood count were assessed for complications.

**Figure 1:**
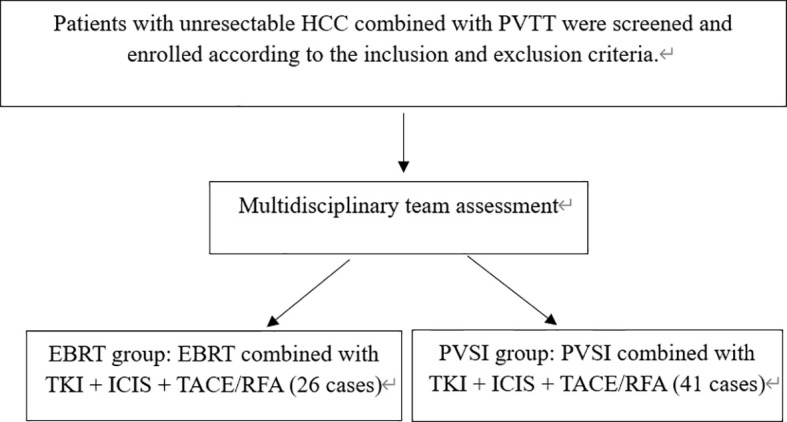
Patient selection flow chart.

Systemic therapy included TKIs (lenvatinib, donafenib, or sorafenib) administered per guidelines, paused three days before and after intervention. ICIs (camrelizumab, tislelizumab, or sintilimab) were infused at 200 mg every three weeks. All patients received at least one cycle (3–4 weeks) of systemic therapy with TKI plus ICI before local treatment (EBRT or PVSI), ensuring an initial systemic response before commencing local intervention. Commonly used first-line TKI combined with ICI regimens include "Lenvatinib + Tislelizumab", "Sintilimab + Sorafenib", and "Apatinib + Camrelizumab". Discontinue treatment upon disease progression, intolerable adverse reactions, or patient withdrawal of consent. Adjuvant TACE or RFA was completed within one month post-PVSI or EBRT. For patients with hepatocellular carcinoma presenting with portal vein tumor thrombosis, the choice between external beam radiotherapy and portal vein stent implantation depends on specific clinical indications, tumor anatomy, and hepatic functional reserve. This determination was made following evaluation by a multidisciplinary team in accordance with established consensus guidelines. Such a collaborative decision-making approach reflects our institutional standard of care. All patients receive comprehensive counseling regarding the potential benefits and risks of each treatment option, tailored to their individual disease status. The final treatment plan incorporates both the multidisciplinary team’s recommendation and the patient’s fully informed consent. (**Figure 1**).

### 2.3 Data collection and monitoring

We recorded treatment parameters (EBRT dose, seed activity) and baseline characteristics (age, HBV status, cirrhosis, Child-Pugh score, PVTT grade, AFP levels). Follow-up assessments occurred monthly after treatment initiation, with tri-monthly CT scans and blood tests measuring tumor biomarkers and hepatic function; MRI supplemented these evaluations when clinically indicated. Outcomes included tumor response, progression-free survival (PFS), overall survival (OS), objective response rate (ORR), disease control rate (DCR), and adverse events (AE). PFS spanned from treatment commencement to disease progression or death, while OS extended from treatment initiation to death. ORR quantified patients achieving partial or complete responses (PR/CR), whereas DCR encompassed those with stable disease (SD), PR, or CR. Tumor response adhered to the modified Response Evaluation Criteria in Solid Tumors (mRECIST) (24). Adverse events were graded per the National Cancer Institute’s Common Terminology Criteria for Adverse Events (NCI-CTCAE) (25) version 5.0.

### 2.4 Statistical analysis

IBM Corp.’s SPSS v27.0 was used to analyze the data. Means ± standard deviation (SD) and percentages (%) were used for continuous and categorical data, respectively. PFS and OS were estimated using Kaplan-Meier curve analysis and compared with the log-rank test. Cox regression was performed as a univariate analysis. Subgroup analysis: Treatment (with or without systemic therapy) and PVTT grade (VP2/3/4) were stratified.

## 3 Results

### 3.1 Features of the patient

This retrospective study analyzed 67 HCC patients with PVTT (**Table 1**). The cohort comprised 41 patients receiving portal vein stent implantation combined with TACE/RFA and TKI+ICIs, and 26 patients treated with external beam radiation (EBRT) plus TACE/RFA and TKI+ICIs; both groups exhibited similar age distributions and male predominance. The baseline characteristics, including ECOG scores, Child-Pugh classification, AFP levels, PVTT severity, tumor type, and the distribution of maximum tumor diameters, were similar across all groups. No statistically significant differences were observed among the groups (P > 0.05), confirming their comparability. The EBRT group received a median radiation dose of 54.0 ± 1.0 Gy (range: 50–60 Gy). No seed migration occurred in the PVSI group, which had a median implantation of 35.0 ± 11.5 125I seeds (range: 25-60). RFA treated 88 lesions (47 in EBRT, 41 in PVSI), while 136 TACE procedures were performed across 67 patients (64 in EBRT, 72 in PVSI). Combined TKI+ICIs therapy averaged 3.2 ± 1.8 cycles (range: 2-5) in the EBRT group and 2.8 ± 1.5 cycles (range: 1-4) in the PVSI group. The distant metastasis patterns of the two groups were largely similar, with the lungs, bones, and retroperitoneal lymph nodes serving as the primary sites.

**Table 1:** Patient demographics and baseline characteristics (n%).

Variables	Group PVSI (n=41)	Group EBRT (n=26)	P Value
Sex			1
Male	36 (87.8)	23 (88.5)	
Female	5 (12.2)	3 (11.5)	
Age(years)			1
>60	8 (19.5)	5 (19.2)	
≤60	33 (80.5)	21 (80.8)	
ECOG Score			0.658
0-1	37 (90.2)	23 (88.5)	
2	4 (9.8)	3 (11.5)	
CNLC stage			0.378
IIIa	24 (58.5)	18 (69.2)	
IIIb	17 (41.5)	8 (30.8)	
Child-Pugh class			0.727
A	36 (87.8)	22 (84.6)	
B	5 (12.2)	4 (15.4)	
AFP (ng/ml)			0.87
≥400	26 (63.4)	17 (65.4)	
<400	15 (36.6)	9 (34.6)	
tumor size (cm)			0.245
≥5	38 (92.7)	21 (80.8)	
<5	3 (7.3)	5 (19.2)	
Extrahepatic metastasis			1
Lung	7 (41.2)	3 (37.5)	
Bone	5 (29.4)	3 (37.5)	
Retroperitoneal	5 (29.4)	2 (25.0)	
Portalhypertension			0.765
Absent	33 (80.5)	20 (76.9)	
Present	8 (19.5)	6 (23.1)	
Systemic therapy			0.642
TKI+ICIs	32 (78)	19 (73.1)	
None	9 (22)	7 (26.9)	
Portal vein tumor thrombus grading			0.205
VP1	0	0	
VP2	14 (34.1)	13 (50)	
VP3	20 (48.8)	7 (26.9)	
VP4	7 (17.1)	6 (23.1)	
Local interventional therapy			0.057
RFA(lesions)	41 (46.6)	47 (53.4)	
TACE (cases)	72 (52.9)	64 (47.1)	

ECOG, Eastern Cooperative Oncology Group; CNLC, Chinese Liver Cancer Staging System; AFP, alpha-fetoprotein; Child-Pugh classification, liver function assessment; TKI, Tyrosine Kinase Inhibitor; ICIs, Immune Checkpoint Inhibitors; RFA, Radiofrequency Ablation; TACE, Transarterial Chemoembolization; VP, VenoPortal.

### 3.2 Survival analysis

**Figure 2:**
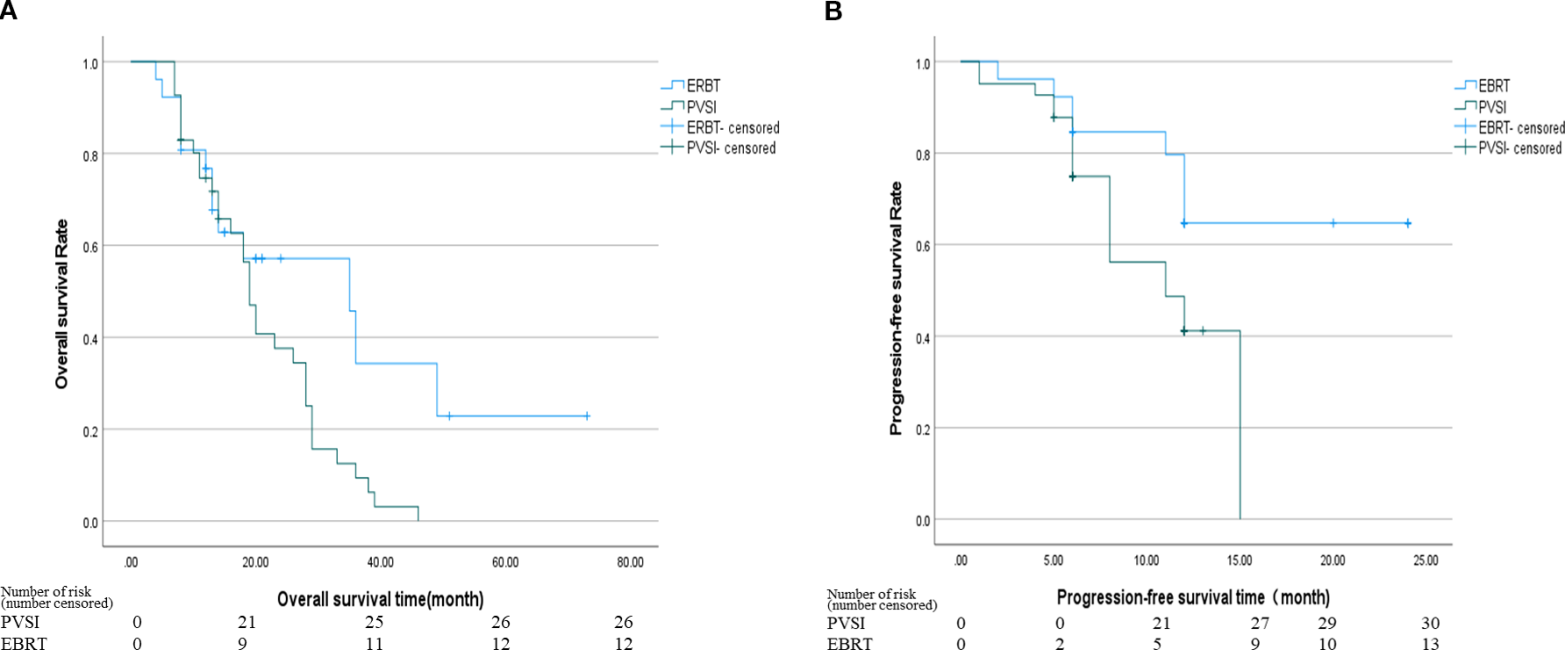
**(A)** The median OS was 35 months (95% CI, 14.5-55.5) in the EBRT group and 19 months (95% CI, 16.9-21.1) in the PVSI group (p = 0.044). **(B)** The median PFS was not reached in the EBRT group and 11 months (95% CI, 6.2-15.8) in the PVSI group (p = 0.037).

**Figure 3:**
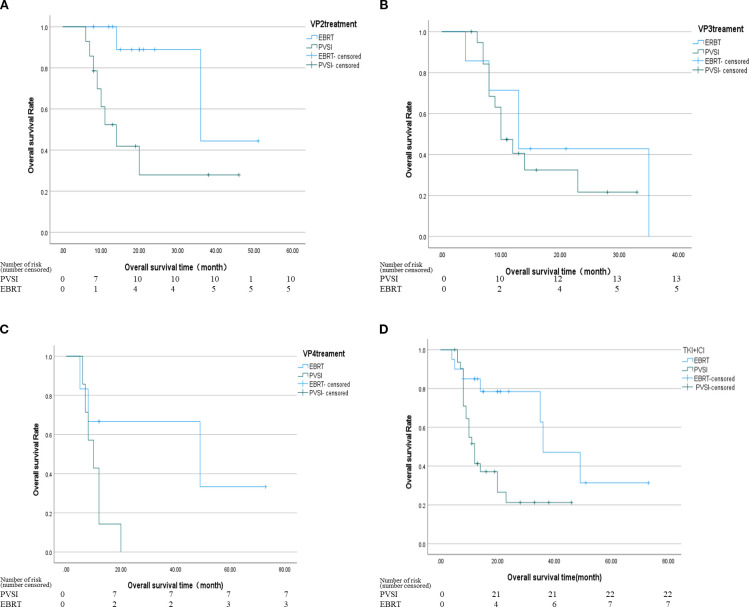
**(A)** The median OS was 36 months (95% CI, 5.1-66.9) in the EBRT group and 14 months in the PVSI group (95% CI, 8.0-20.0) (p = 0.017); **(B)** The median OS was 13 months (95% CI, 6.6-19.4) in the EBRT group and 10 months (95% CI, 7.0-13.0) in the PVSI group (p = 0.507); **(C)** The median OS was 49 months (95% CI, 0.0-110.4) in the EBRT group and 10 months (95% CI, 4.9-15.1) in the PVSI group (p = 0.066); **(D)** The median OS was 36 months (95% CI, 20.1-51.9) in the EBRT group and 12 months (95% CI, 9.7-14.3) in the PVSI group (p = 0.005).

**Table 2** demonstrates superior short-term efficacy in the EBRT group compared to PVSI (median follow-up 21.0 months; range 4–72 months), with significantly higher 6-month ORR (38.5% vs. 24.4%, p=0.028) and DCR (84.6% vs. 58.5%, p=0.025). **Figures 2A, B** reveal that the median OS was 35 months (95%CI, 14.5-55.5 ) in group EBRT and 19 months in group PVSI (95%CI, 16.9-21.1) (p = 0.044), the median PFS was not reached in the EBRT group and 11 months (95% CI, 6.2-15.8) in the PVSI group (p = 0.037). Multivariate analysis confirmed that EBRT treatment (HR=2.247, 95% CI, 1.090–5.404, P=0.030) and AFP < 400 ng/ml (HR=0.329, 95% CI, 0.137-0.791, P=0.013) were independent predictors of overall survival (**Table 3**). Further subgroup analysis revealed a particularly pronounced survival benefit from EBRT in patients with VP2-type portal vein tumor thrombus and in those treated with TKI plus ICI therapy. For patients with VP2 thrombus, median overall survival was 36 months (95% CI: 5.1–66.9) in the EBRT group compared to 14 months (95% CI: 8.0–20.0) in the PVSI group (p = 0.017). Among those receiving TKI and ICI combination therapy, median overall survival was 36 months (95% CI: 20.1–51.9) with EBRT versus 12 months (95% CI: 9.7–14.3) with PVSI (p = 0.005) (**Figures 3A–D**).

**Table 2:** Patient response according to mRECIST outcomes (n%).

Time	Response	Group PVSI(n=41)	Group EBRT(n=26)	P Value
3 months	CR	0 (0.0)	4 (15.4)	
	PR	10 (24.4)	6 (23.1)	
	SD	19 (46.3)	14 (53.8)	
	PD	12 (29.3)	2 (7.7)	
	ORR	25 (24.4)	10 (38.5)	0.072
	DCR	29 (70.7)	24 (92.3)	0.34
6 months	CR	0 (0.0)	4 (15.4)	
	PR	6 (14.6)	6 (23.1)	
	SD	18 (43.9)	12 (46.2)	
	PD	16 (36.6)	5 (19.2)	
	ORR	6 (14.6)	10(38.5)	0.028
	DCR	24(58.5)	22 (84.6)	0.025

CR, complete response; DCR, disease control rate; PD, progressive disease; PR, partial response; SD, stable disease. ORR = (CR + PR)/n. DCR = (CR + PR + SD)/n.

**Table 3:** Univariate and Multivariate Analysis of Factors Associated with Overall Survival.

Variables	Univariate		Multivariate	
	HR (95%CI)	P Value	HR(95%CI)	P Value
Age, <60 vs. ≫60	0.859 (0.609-1.211)	0.386	0.573 (0.254-1.297)	0.182
Portal hypertension, absent vs. present	0.852 (0.579-1.253)	0.416	0.523 (0.223-1.227)	0.136
Sex, man vs. woman	1.077 (0.698-1.622)	0.736	2.185 (0.776-6.154)	0.139
Classification of portal vein tumor thrombus, VP2 vs. VP3 vs. VP4	1.172 (0.471-1.107)	0.457	0.514 (0.210-1.260)	0.146
Child-Pugh class, A vs. B	1.124 (0.748-1.689)	0.574	0.889 (0.321-2.461)	0.821
Tumor size, ≪5 vs. >5	0.814 (0.568-1.165)	0.260	0.835 (0.365-1.913)	0.670
CNLC stage, IIIa vs. IIIb	1.011 (0.742-1.379)	0.944	0.970 (0.468-2.012)	0.935
AFP, <400 vs. ≥400 ng/ml	0.701 (0.496-0.990)	0.044	0.329 (0.137-0.791)	**0.013**
Treatment, EBRT vs. PVSI	2.340 (1.187-4.613)	0.014	2.247 (1.090-5.404)	**0.030**
Interventional therapy, TACE vs RFA	0.284(0.111-0.725)	0.080	0.354 (0.120-1.045)	0.060
TKI+ICI vs None TKI+ICI	0.496(0.227-1.080)	0.077	0.457 (0.185-1.132)	0.091

CI, confidence interval; HR, hazard ratio. A p-value of 0.05 was considered to indicate statistical significance.

### 3.3 Safety analysis

The treatment did not induce severe side effects such as radiation hepatitis, liver abscess, acute liver failure, or abdominal bleeding. The most frequent grade 1–2 adverse events were fever, fatigue, nausea, vomiting, abdominal discomfort, rash, abnormal liver function, and bone marrow suppression. Leukopenia occurred more often in the EBRT group than in the PVSI group (46.2% vs. 7.3%, P < 0.001). For patients presenting with grade 1–2 hematologic toxicity, EBRT treatment was not routinely interrupted. All patients received prophylactic supportive care, including oral leukocyteelevating agents such as Leukine. EBRT or TKI treatment was suspended only in cases of grade 3 or higher hematologic toxicity, with adjunct use of recombinant human granulocyte colony-stimulating factor (G-CSF). No patient in this study permanently discontinued treatment due to hematologic toxicity. Conversely, grade 1–2 AST elevation was more prevalent in the PVSI group (70.7% vs. 38.5%, P = 0.009), suggesting greater liver dysfunction in these patients. Grade 3–4 adverse events were rare in both groups, though the PVSI group exhibited nearly 20% rates of TBIL and ALB abnormalities. All adverse events were resolved with symptomatic management. **Table 4** summarizes the adverse reactions observed in both cohorts.

**Table 4:** Classification and incidence of treatment-related adverse events (n%).

Adverse events	Grading	Group PVSI	Group EBRT	P Value
Abdominal pain	1-2	5 (12.2)	4 (15.4)	0.727
	3-4	0 (0)	0 (0)	
Leukopenia	1-2	3 (7.3)	12 (46.2)	<0.001
	3-4	1 (2.4)	0 (0)	
Thrombopenia	1-2	5 (12.2)	8 (30.8)	0.061
	3-4	2 (4.9)	3 (11.5)	0.369
Skin rash	1-2	5 (12.2)	7 (26.9)	0.191
	3-4	1 (2.4)	0 (0)	
Hepatic-dysfunction				
TBIL	1-2	13 (31.7)	7 (26.9)	0.677
	3-4	9 (22)	2 (7.7)	0.181
ALT	1-2	8 (19.5)	4 (15.4)	0.753
	3-4	1 (2.4)	0 (0)	
AST	1-2	29 (70.7)	10 (38.5)	0.009
	3-4	3 (7.3)	2 (7.7)	1
ALB	1-2	22 (53.7)	10 (38.5)	0.225
	3-4	8 (19.5)	2 (7.7)	0.294

TBIL, total bilirubin; ALT, alanine aminotransferase; AST, aspartate aminotransferase; ALB, albumin.

## 4 Discussion

This study compared the safety and effectiveness of external Beam Radiotherapy (EBRT) versus Portal Vein Stent Implantation (PVSI) when combined with local interventional therapy and TKI plus ICIs in patients with hepatocellular carcinoma (HCC) and portal vein tumor thrombus (PVTT) in a real-world setting. The findings showed that the EBRT group experienced significantly greater survival benefits than the PVSI group: the 6-month objective response rate (ORR) and disease control rate (DCR) were notably higher (38.5% vs. 24.4%; 84.6% vs. 58.5%), median overall survival (OS) nearly doubled (35 months vs. 19 months, P = 0.044), and the progression-free survival (PFS) of the EBRT group did not reach the endpoint. Compared with the EBRT group in this study, Sahai et al. (26) reported a median OS of 10.9 months for radiotherapy combined with systemic therapy and DEB-TACE, and observed significantly prolonged survival among patients with PVTT remission, indicating that radiotherapy effectively controls PVTT. These findings offer external validation for the survival outcomes observed in the EBRT group of this study. The results are consistent with previous findings by Tang (27), who demonstrated the therapeutic benefits of radiotherapy for HCC with PVTT. Their study revealed that tumor antigen exposure induced inflammatory responses, modulated immune markers, and promoted tumor necrosis. However, our results differ from those reported by Tan et al. (28). Their subgroup analysis demonstrated superior overall survival (11.7 vs. 7.6 months, p < 0.001) for VP4-type PVTT patients receiving PVSI compared to EBRT, supporting the conclusion that PVSI + TACE yields better survival outcomes for PVTT HCC patients than RT + TACE. One possible explanation is that most PVTT cases in this study were of the VP2 type, whereas Tan’s study mostly involved VP4 type PVTT (50 out of 53 cases). This further suggests that PVSI can quickly open blood vessels in fully blocked VP4 thrombi, while EBRT can precisely target tumors in VP2 thrombi with partial blood flow. Additionally, this study combined TACE/RFA and (TKI+ICIs), whereas Tan’s study used only TACE. The increased effectiveness of TKI+ICIs might partly explain the survival benefit observed in the EBRT group. Furthermore, the use of TKI+ICIs for liver cancer treatment may have been enhanced by irradiation (27), as Tan’s research did not incorporate systemic treatments beyond that, which could limit long-term effectiveness. The third reason is that this study employed a conventional single-row particle chain, whereas Tan et al. used a four-row I-125 particle scaffold offering 360 coverage. This configuration may promote rapid vascular expansion. Consequently, for patients with VP4-type tumors, the PVSI approach did not yield superior outcomes compared to EBRT in this investigation.

According to the study’s subgroup analysis, the median overall survival (OS) for patients with VP2 type tumor thrombus following EBRT was 36 months, compared to 14 months in the PVSI group (P = 0.017), indicating that tumor thrombus classification is a significant predictor of success. Radiotherapy can more effectively treat the local lesion and preserve liver function in cases of VP2 type tumor thrombus, which involves the secondary branch of the portal vein and does not completely block the main blood flow. However, VP3/VP4 classifications may better guide goal-oriented PVSI due to their association with more extensive tumor thrombus invasion (29). This stratified outcome emphasizes the need for customized care, and moving forward, a type-specific approach based on imaging characteristics should be developed. Given the relatively small sample size of the VP3/VP4 subgroup, this analysis remains exploratory, and its findings require validation through larger future studies.

The EBRT group exhibited significantly higher hematological toxicity, with grade 1–2 leukopenia occurring in 46.2% of patients versus 7.3% in the PVSI group (P < 0.001), reflecting bone marrow suppression and underscoring the importance of enhanced hematopoietic monitoring. Liver function abnormalities were more pronounced in the PVSI group, where AST levels rose in 70.7% of cases compared to 38.5% (P = 0.009), likely due to localized particle radiation and mechanical injury to hepatic sinusoidal endothelial cells during stent placement. These findings corroborate the mechanisms reported by Tan et al. (28, 30). Among the grade 3 adverse events, the most common were thrombocytopenia, elevated bilirubin, elevated AST, and elevated ALB. The incidence of each event, however, remained below 20%. Both groups showed relatively low rates of severe adverse events (》grade 3). There was no statistically significant difference, suggesting that the toxicity profiles of both treatments align with their known effects and are safe and manageable within the study cohort.

Multivariate analysis identified EBRT treatment (HR=2.247) and AFP < 400 ng/ml (HR = 0.329) as independent prognostic factors. Lower AFP levels correlated with improved response to comprehensive treatment, consistent with its established role as a biomarker for hepatocellular carcinoma aggressiveness. These findings reinforce the utility of AFP in pretreatment risk stratification and corroborate the PVTT prognostic model reported by Peng et al. (31). In addition to the prognostic relevance of alpha-fetoprotein (AFP) that we observed in our research, there is also a growing interest in incorporating serum biomarkers such as AFP and des-gamma-carboxy prothrombin (DCP) into the diagnostic algorithms for screening and monitoring hepatocellular carcinoma (HCC), especially in high-risk populations. For these patients, especially those with markedly elevated AFP and DCP alongside vascular invasion or high risk of early recurrence, more aggressive local therapies like external beam radiotherapy (EBRT) may be warranted over transarterial chemoembolization (TACE) or other interventional approaches. Such cases often respond poorly to conventional interventional treatments, whereas EBRT can achieve broader local control, particularly for tumors in challenging locations or with extensive vascular involvement (32).

## 5 Limitations and prospects

The study has several limitations, including potential selection bias due to its retrospective design. The small sample size (n = 67) may limit the reliability of subgroup analyses, particularly since the VP3/VP4 subgroup did not exhibit significant differences. The observation period should be extended, since the median PFS in the EBRT group did not reach the threshold, suggesting insufficient follow-up. Furthermore, the impact of radiotherapy techniques such as SBRT on survival has not been evaluated. The analysis did not account for subsequent second or third-line treatments, which may confound the attribution of survival benefits. Another constraint is the limited number of patients in the VP2 subgroup. These results should thus be interpreted as preliminary and warrant validation in larger prospective studies. In this study, the absolute number of grade 3 or higher adverse events was relatively small. Further confirmation of these findings is needed in a larger cohort in the future. Sensitivity analysis was not conducted to assess the potential impact of unmeasured confounding factors. While this study did not investigate novel mechanisms or biomarkers, our clinically derived findings illuminate a path for future translational research. Subsequent studies could explore which biomarkers—such as specific genetic mutations or features of the immune microenvironment — might identify patient subgroups most likely to benefit from EBRT- or PVSI-based combination strategies.

## 6 Conclusion

Hepatocellular carcinoma (HCC) patients with portal vein tumor thrombus (PVTT) exhibit significantly improved survival when treated with combined external beam radiotherapy (EBRT), local interventional procedures, and tyrosine kinase inhibitors plus immune checkpoint inhibitors (TKI+ICIs). Patients with VP2-type PVTT achieve a median survival of nearly three years with this approach. Clinicians must remain vigilant regarding hematological toxicity. Clinical decision-making should incorporate four-dimensional assessments, including bone marrow tolerance, hepatic functional reserve, AFP levels, and tumor thrombus classification. While portal vein stent implantation (PVSI) serves as a crucial alternative for patients with compromised liver function or VP3/VP4-type PVTT, EBRT remains the preferred option for VP2-type cases with preserved bone marrow function. Further investigations should prioritize refining radiation techniques, optimizing dosing protocols, developing advanced particle stent materials, and implementing biomarker-guided personalized therapies.

## Data Availability

The datasets presented in this article are not readily available because patient-identifiable clinical records, imaging data, and laboratory results are restricted by ethical protocols and accessible only to the research team. Requests to access the datasets should be directed to luowenping, luowenping0775@16.com.

## References

[1] International Agency for Research on Cancer. Global cancer observatory: liver cancer fact sheet 2023. Lyon, France: World Health Organization (2023).

[2] Ellis L Canchola AJ Spiegel D Ladabaum U Haile R Gomez SL. Trends incancer survival by health insurance status in california from 1997 to 2014. JAMA Oncol. (2018) 4(3):317–23. doi: 10.1001/jamaoncol.2017.3846, PMID: 29192307 PMC5885831

[3] Soin A Lesurtel M Bhangui P Cocchi L Bouattour M Clavien PA. Are patients with hepatocellular carcinoma and portal vein tumor thrombosis candidates for liver transplantation? J Hepatol. (2023) 78:1124–9. doi: 10.1016/j.jhep.2023.03.032, PMID: 37208099

[4] Reig M Forner A Rimola J Ferrer-Fàbrega J Burrel M Garcia-Criado Á. BCLC strategy for prognosis prediction and treatment recommendation: The 2022 update. J Hepatol. (2021) 76(3):681–93. doi: 10.1016/j.jhep.2021.11.018, PMID: 34801630 PMC8866082

[5] National Health Commission of the People's Republic of China. Guidelines for the diagnosis and treatment of primary liver cancer (2024 edition). J Clin Hepatol. (2024) 40:893–918. doi: 10.21147/j.issn.1000-9604.2019.02.02, PMID: 31156298 PMC6513740

[6] Li S Guo JH Lu J Wang C Wu H Wang H. I125 irradiation stent for treatment of hepatocellular carcinoma with portal vein thrombosis: A meta-analysis. Cancer Radiother. (2021) 25:340–9. doi: 10.1016/j.canrad.2020.12.003, PMID: 33455874

[7] Zhang ZH Zhang W Gu JY Liu QX Ma JQ Liu LX. Treatment of hepatocellular carcinoma with tumor thrombus using iodine-125 seed strand implantation and transarterial chemoembolization: a propensity-score analysis. J Vasc Interv Radiol. (2018) 29:1085–93. doi: 10.1016/j.jvir.2018.02.013, PMID: 29754851

[8] Hong D Zhou Y Wan X Su H Shao H. Brachytherapy with Iodine-125 seeds for treating portal vein-branch tumor thrombus in patients with hepatocellular carcinoma. BMC Cancer. (2021) 21:1020. doi: 10.1186/s12885-021-08680-0, PMID: 34521375 PMC8439081

[9] Lin J Jiang H Yang W Jiang N Zheng Q Huang N. Predictive factors for benefit from iodine-125 brachytherapy in treating hepatocellular carcinoma with portal vein tumor thrombosis. Brachytherapy. (2019) 18:233–39. doi: 10.1016/j.brachy.2018.10.002, PMID: 30467014

[10] Ji X Xu Z Sun J Li W Duan X Wang Q. Lenvatinib with or without stereotactic body radiotherapy for hepatocellular carcinoma with portal vein tumor thrombosis: a retrospective study. Radiat Oncol. (2023) 18:101. doi: 10.1186/s13014-023-02270-z, PMID: 37308914 PMC10259021

[11] Li G Shu B Zheng Z Yin H Zhang C Xiao Y. Safety and efficacy of radiotherapy combined with lenvatinib plus PD-1 inhibitors as neo-adjuvant therapy in hepatocellular carcinoma with portal vein thrombus: protocol of an open-label, single-arm, prospective, multi-center phase I trial. Front Oncol. (2022) 12:1051916. doi: 10.3389/fonc.2022.1051916, PMID: 36505833 PMC9730694

[12] Wang K Xiang YJ Yu HM Cheng Y-Q Liu Z-H Zhong J-Y. Intensity-modulated radiotherapy combined with systemic atezolizumab and bevacizumab in the treatment of hepatocellular carcinoma with extrahepatic portal vein tumor thrombus: A preliminary multicenter single-arm prospective study. Front Immunol. (2023) 14:1107542. doi: 10.3389/fimmu.2023.1107542, PMID: 36875125 PMC9978499

[13] Zhang ZH Hou SN Yu JZ Zhang W Ma J-Q Yang M-J. Combined iodine-125 seed strand, portal vein stent, transarterial chemoembolization, lenvatinib, and anti-PD-1 antibodies therapy for hepatocellular carcinoma and Vp4 portal vein tumor thrombus: A propensity-score analysis. Front Oncol. (2023) 12:1086095. doi: 10.3389/fonc.2022.1086095, PMID: 36741718 PMC9893110

[14] Sun H Zhang M Liu R Liu Y Hou Y Wu C. Endovascular implantation of 125I seed combined with transcatheter arterial chemoembolization for unresectable hepatocellular carcinoma. Future Oncol. (2018) 14:1165–76. doi: 10.2217/fon-2017-0354, PMID: 29334777

[15] European Association for the Study of the Liver. EASL Clinical Practice Guidelines: Management of hepatocellular carcinoma. J Hepatol. (2018) 69:182–236. doi: 10.1016/j.jhep.2018.03.019, PMID: 29628281

[16] Expert Committee of Guidelines for the Diagnosis and Treatment of Primary Liver Cancer. Guideline for the diagnosis and treatment of primary liver cancer (2024 edition). J Chin J Clin Med. (2024) 31(2).

[17] European Association for the Study of the Liver. EASL Clinical Practice Guidelines on the management of hepatocellular carcinoma. J Hepatol. (2025) 82:315–74. doi: 10.1016/j.jhep.2024.08.028, PMID: 39690085

[18] Expert committee of the guideline for the diagnosis and treatment of primary liver cancer (2024 edition). Guideline for the diagnosis and treatment of primary liver cancer. J Chin J Clin Med. (2024) 31(2).

[19] Azam F Latif MF Farooq A Tirmazy SH AlShahrani S Bashir S. Performance status assessment using ECOG (Eastern Cooperative Oncology Group) score for cancer patients by oncology healthcare professionals. Case Rep Oncol. (2019) 12:728–36. doi: 10.1159/000503095, PMID: 31616281 PMC6792426

[20] Shi J Lai ECH Li N Guo W-X Xue J Lau WY. Surgical treatment of hepatocellular carcinoma with portal vein tumor thrombus. Ann Surg Oncol. (2010) 17:2073–80. doi: 10.1245/s10434-010-0940-4, PMID: 20131013

[21] Radiation Oncology Branch of the Chinese Medical Association Expert Committee on Liver Cancer and Digestive System of China Institute of Biomedical Engineering Liver Cancer Research Group of Radiation Oncology Branch of China Research Hospital. Consensus on radiation therapy for primary liver cancer in 2016. Chin J Radiat Oncol. (2016) 25:1141–50.

[22] Pan CC Kavanagh BD Dawson LA Li XA Das SK Miften M. Radiation-associated liver injury. Int J Radiat Oncol Biol Phys. (2010) 76:S94–S100. doi: 10.1016/j.ijrobp.2009.06.092, PMID: 20171524 PMC4388033

[23] Marks LB Yorke ED Jackson A Ten Haken RK Constine LS Eisbruch A. Use of normal tissue complication probability models in the clinic. Int J Radiat Oncol Biol Phys. (2010) 76:S10–9. doi: 10.1016/j.ijrobp.2009.07.1754, PMID: 20171502 PMC4041542

[24] Lencioni R Llovet JM. Modified RECIST (mRECIST) assessment for hepatocellular carcinoma. Semin Liver Dis. (2010) 30:52–60. doi: 10.1055/s-0030-1247132, PMID: 20175033 PMC12268942

[25] CTCAE v5.0 grading . Available online at: https://ctep.cancer.gov/protocolDevelopment/electronicapplications/ctc.htmctc_50 (accessed on May 23, 2025).

[26] Sahai P Yadav H P Choudhury A Shasthry S M Jindal A Mall A. Outcomes with radiotherapy in multimodality treatment for hepatocellular carcinoma with portal vein tumour thrombosis. BJR Open. (2025) 7(1):tzaf002. doi: 10.1093/bjro/tzaf002, PMID: 40093581 PMC11909637

[27] Tang C He Q Feng J Liao Z Peng Y Gao J. Portal vein tumor thrombosis radiotherapy improves the treatment outcomes of immunotherapy plus bevacizumab in hepatocellular carcinoma: a multicenter real-world analysis with propensity score matching. Front Immunol. (2023) 14:1254158. doi: 10.3389/fimmu.2023.1254158, PMID: 37928530 PMC10620737

[28] Tan Z Lu J Zhu G Chen L Wang Y Zhang Q. Portal vein irradiation stent plus chemoembolization versus external radiotherapy plus chemoembolization in hepatocellular carcinoma with portal vein tumor thrombus: a retrospective study. Cardiovasc Intervent Radiol. (2021) 44:1414–22. doi: 10.1007/s00270-021-02889-z, PMID: 34131776

[29] Huang J Cai M Huang W Guo Y Zhou J Liang L. Transarterial chemoembolization combined with sorafenib and iodine-125 seed brachytherapy for hepatocellular carcinoma with portal vein tumor thrombus: a retrospective controlled study. Chin Med J (Engl). (2022) 135:113–5. doi: 10.1097/CM9.0000000000001537, PMID: 34507316 PMC8850867

[30] McConnell MJ Kostallari E Ibrahim SH Iwakiri Y. The evolving role of liver sinusoidal endothelial cells in liver health and disease. Hepatology. (2023) 78:649–69. doi: 10.1097/HEP.0000000000000207, PMID: 36626620 PMC10315420

[31] Wang XH Liu QB Xiang CL Woldesenbet S Khalil M Sahara K. Multi-institutional validation of novel models for predicting the prognosis of patients with large hepatocellular carcinoma. Int J Cancer. (2021) 149:127–38. doi: 10.1002/ijc.33516, PMID: 33586134

[32] Shahini E Pasculli G Solimando A G Tiribelli C Cozzolongo R Giannelli G. Updating the clinical application of blood biomarkers and their algorithms in the diagnosis and surveillance of hepatocellular carcinoma: A critical review. Int J Mol Sci. (2023) 24(5):4286. doi: 10.3390/ijms24054286, PMID: 36901717 PMC10001986

